# Correlations of Equilibrium Properties and Electronic Structure of Pure Metals

**DOI:** 10.3390/ma12182932

**Published:** 2019-09-11

**Authors:** Jianhong Dai, Dongye He, Yan Song

**Affiliations:** 1 School of Materials Science and Engineering, Harbin Institute of Technology at Weihai, 2 West Wenhua Road, Weihai 264209, China; 2 School of Materials Science and Engineering, Jilin University, 5988 Renmin Street, Changchun 130025, China

**Keywords:** first principles calculations, metals, electronic parameters, elastic properties

## Abstract

First principles calculations were carried out to study the equilibrium properties of metals, including the electrons at bonding critical point; *e_bcp_*; cohesive energy; *E_coh_*; bulk modulus; *B*; and, atomic volume; *V*. 44 pure metals, including the *s* valence (alkali), *p* valence (groups III to V), and *d* valence (transition) metals were selected. In the present work, the electronic structure parameter *e_bcp_* has been considered to be a bridge connecting with the equilibrium properties of metals, and relationships between *e_bcp_* and equilibrium properties (*V*; *E_coh_*; and *B*) are established. It is easy to estimate the equilibrium properties (*E_coh_*; *V*, and *B*) of pure metals through proposed formulas. The relationships that were derived in the present work might provide a method to study the intrinsic mechanisms of the equilibrium properties of alloys and to develop new alloys.

## 1. Introduction

The design of materials with desirable properties associating with computational simulations has currently become a normal approach. It is well known that the physical properties are controlled by the bonding between atoms, which is strictly determined by the electronic structure of the materials. Although the mechanical properties of materials, such as the elastic modulus, strength, toughness, and ductility are macro-properties, they are all related to the breaking and reforming of interatomic bonds, and therefore determined by the characteristics of electronic structures [1,2,3,4]. The understanding of electronic factors that affect the mechanical properties can boost these search processes. So far, efforts have been made to build a relationship between these atomistic-scale parameters and the macroscopic mechanical behaviors, such as between bulk modulus of metals and their lattice volume [5]. It is very convenient to evaluate the bulk modulus of materials by fitting the energy-strain or stress-strain curve that was obtained via first principles calculations [6,7]. Metal crystals are combined by the attractions of Coulomb forces between the metal cations and electrons. The attractions have no directions, the closer between the metal cations, the stronger of the attractions are. The energy of crystals will be gradually increased with the compression of crystal cell (such as under the hydrostatic pressures). In general, the electrons in metal crystals move freely, and the repulsive force between them can resist the compression of crystals due to the affection of Pauli’s exclusion principle. Therefore, the distributions of electrons in metal are correlated with the bulk modulus of metals.

Many methods were proposed to analyze the distributions of electrons in materials, such as Mulliken population analysis [8], Natural Population Analysis (NPA) [9], and Topology analysis, the Atoms In Molecules theory (AIM), as proposed by Bader et al. [10,11,12,13]. The Mulliken population analysis is a familiar method for analyzing the electrons with an atom, which simply separate the electrons in crystal and help us to estimate the bonding properties of atom in molecule. However, it is arbitrary and strongly dependent on the employed particular basis set. The NPA of electron density is based on the orthonormal natural atomic orbitals [9]. It improves the numerical stability and overcomes the basis set dependence problem of Mulliken population analysis, which is better than the Mulliken method in describing the electron distribution in metals. In AIM [10,11,12,13], molecules are divided into atoms by the gradient of electron density. The gradient of electron density has no flux in the surfaces of atoms. The critical points can be found by calculating the Laplacian value of electron density. The AIM theory has been successfully applied to describe the bonding properties between the atoms in molecule. The critical points are classified by the eigenvalues of the Hessian matrix at the point. The bonding critical point (*bcp*) is a first-order saddle point in the electron density distribution. The Hessian matrix at a bonding critical point has two negative and one positive eigenvalues, which can be denoted as (3, −1). The bonding strength between atoms can be explained by the charge distributions.

There are some works that have been concentrated in building the relationship between the charge distributions and the bulk properties of materials. Segall et al. established the correlations of overlap population with covalence of bonding and bond strength, and of the effective valence charge with ionicity of bonding by means of Mulliken analysis [14], and found that the bulk modulus increases with the overlap populations [14]. Al-Douri et al. built an empirical model between the bulk modulus and the charge density in semiconductors, where the bulk modulus relates with the area of the cation side of the total valence pseudo-charge density [15]. Miedema et al. introduced an empirical relationship between the bulk modulus (*B*) of several pure metals and electronic density *n_WS_* at the boundary of the Wigner–Seitz cell: *n_WS_* = 0.82 × (*B/V_m_*)^1/2^, where *V_m_* is atomic volume [16]. Cheng et al. calculated the values of *n_WS_* of fcc and bcc metals firstly by first principles calculations [17]. They modified the empirical relationship of Miedema et al. to *B* = (1.487 × 10^8^) *V_m_n*^2^*_WS_*. Although their calculated values of *n_WS_* are consistent well with the values of Miedema et al., it is very hard to evaluate the *n_WS_* values for complex metals or alloys. Li et al. [18,19] derived a simple empirical model for estimating the bulk modulus of binary intermetallic compounds and alloys from the Miedema’s model. They found that the bulk moduli of binary systems can be predicted (with an average error limit of 11%). However, the average numbers of electrons at the boundary of the Wigner–Seitz cell hardly show the detailed bonding information between atoms, and the simple average of electrons might lose some important information, especially for the crystals with different elements.

Besides applying the first principles calculations, Wills et al. predicted the total energy of transition metal as a function of volume and ionic configuration by extending the nearly-free-electron theory to include the effects of transition-metal *d* bands, which provides a qualitative prediction of the elastic and bonding properties of transition metals. They found that the total energy can be used to describe the cohesive and elastic properties of metals. However, their methods included many approximations, such as Thomas–Fermi approximation, and only empty-core pseudo-potentials are considered in treating the conduction electrons [20]. Makino et al. found that the bulk modulus of an elemental substance can be empirically related with the effective pseudopotential radius. Although he attempted to consider the effects of *sd* and *sp* hybridizations in his work, the empirical parameters are, however, different with the different types of metals. There was not a general and effective way to build clear empirical relationship based on this work [21]. Raju et al. [22] studied the pressure derivative of bulk modulus calculated for the entire block of *d*-transition metals while using a modified form of the recipe that was proposed by Wills and Harrison [20] to represent the effective interatomic interaction. A global correlation between the pressure derivative of bulk modulus and the bonding or the interstitial electron density was proposed [22]. Goble et al. [23] established empirical equations between the mineral hardness, bulk modulus, the volumetric cohesive energy (*E_coh_*/*V*), and the hardness. Singh et al. [24] calculated the elastic constants of nine transition metals and four rare-earths and actinides while using the ion–ion interaction. They found that the contributions of volume to bulk modulus varied between 17.1% and 62.4%, which are quite significant and important in quantitatively describing the Cauchy ratio for the considered metals.

So far, the bonding characteristics are generally based on “atomistic level” but not “electronic level”, since the energy, forces, and stress parameters are usually described based on chemical element types and positions of each atom involved in deformation. The lack of fundamental understanding on their electronic features increases the complexity for search in the large composition parameters to design the advanced alloys with improved mechanical properties, especially for multicomponent alloy systems, such as high entropy alloys and many commercial alloys [25,26].

Although many works have been concentrated on studying the bulk modulus of materials, other equilibrium properties (such as volume, cohesive energy, and structural parameters) have been rarely referred. Furthermore, it is important to build the relationships between the electronic structures and the macro properties of materials. Therefore, in this work, the electronic structures and the correlations with equilibrium properties of metals are studied in detail.

## 2. Methodology

First-principles total energy calculations were performed for 44 pure metals with bcc, fcc, and hcp structures within the framework of Kohn−Sham density functional theory (DFT) [27,28,29,30] while using the projector augmented wave (PAW) approach [31,32] for the description of ion-electron interaction, as implemented in the Vienna ab initio simulation package (VASP). Electron exchange–correlation was treated within the generalized gradient approximation (GGA) using the PW91 functional [33] according to the reports of Shang et al [5]. A cutoff energy of 450 eV and a Gaussian smearing method with an energy broadening of 0.15 eV were used throughout. Self-consistent field convergence was considered for a total energy difference of less than 10^−5^ eV between iterations. The conjugate gradient algorithm was used to relax ions and the ionic relaxation was stopped when the forces acting on ions dropped below 0.01 eV/Å. The k-points are carefully checked in the optimization of the lattice structure of pure metals. The optimized structures are well consistent with theoretical calculations [5]. The electronic structures of the optimized structures were recalculated by the full potential linearized augmented plane-wave code WIEN2K [34] under the framework of generalized gradient approximation (PBE-GGA) using the Perdew-Burke-Ernzerhof exchange-correlation potential in order to obtain the values of electrons at the *bcp*. The self-consistency procedure was performed with 2000 *k* points in the irreducible part of the Brillouin zone. The criterion for energy convergence is set to be 0.0001 Ry.

## 3. Results and Discussions

**Figure 1:**
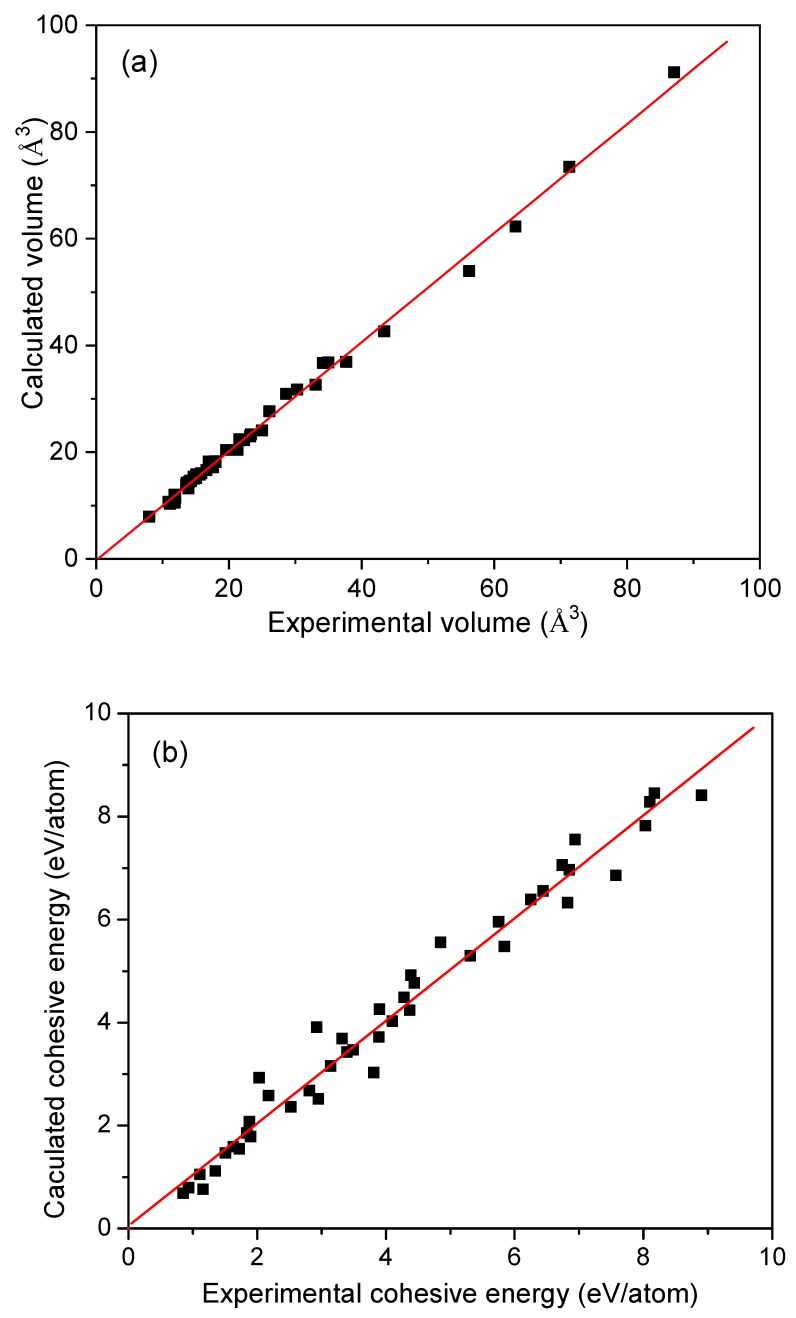
The relationships between calculated values and experimental values of (**a**) atomic volume of metal and (**b**) cohesive energy *E_coh_*.

The phase stability of crystals is evaluated by the cohesive energy defined by:

(1) $$E_{coh}=\frac{N\cdot E_{a}-E_{M}}{N}$$

where *N* is the number of atoms in the unit cell. *E_M_* and *E_a_* denote the energies of crystal and atom, respectively. The energy of metal atom is evaluated by putting it in a 1 × 1 × 1 nm^3^ cell and the calculated total energy of the cell is regarded as the energy of a metal atom. Table 1 shows the evaluated cohesive energies and the experimental values [35] of studied metals. Furthermore, the atomic volume of metal is chosen to examine the accuracy of the calculations by comparing them with experimental measurements, as shown in Figure 1a. A strictly linear relationship, with a slope closing to unity (1.02), between the theoretical and experimental values is illustrated in this figure. Although most of the calculated values are much closed to the experimental values, there are some discrepancies between theoretical and experimental values. Therefore, both the experimental and theoretical values of volumes, cohesive energies and bulk modulus (from Ref. [5]) were used in this work to improve the precision of our empirical formula. 

Besides the above calculations, we also evaluate the numbers of electrons at the *bcp*, *e_bcp_*, of studied metals, which are also listed in Table 1. As mentioned above in AIM theory the *e_bcp_* correlates with the bond interaction between atoms in molecule. In general, the *e_bcp_* of alkali metals are very small, while the values are much larger in transition metals, which might be related with the difference structures of valence electrons in them. The relationships between the *e_bcp_* and micro-properties, such as the cohesive energy, atomic volume of metal, and bulk modulus were further analyzed. 

Shang et al. have calculated the equations of state (EOS) of pure elements, and they found that the PAW-GGA could correctly describe the volume and elastic stiffness constants of most pure elements. However, there are large differences (>3%) for the rare earth elements (Ce, Ac, and Eu), and heavy transition metals (Ag, Au, Pb, Pd, and Pt) [5]. It is worth noting that the van der Waals corrections should be considered for these systems in order to obtain accurate calculation results [36].

The *e_bcp_* is slightly affected by the calculation details, such as the smearing method, k-mesh, and exchange–correlation functionals. The calculated *e_bcp_* of vanadium using PBE-GGA, LSDA, WC-GGA (Wu-Cohen 2006), and PBEsol-GGA (Perdew et al. 2008) are 0.2900, 0.2913, 0.2911, and 0.2914, respectively. 

**Figure 2:**
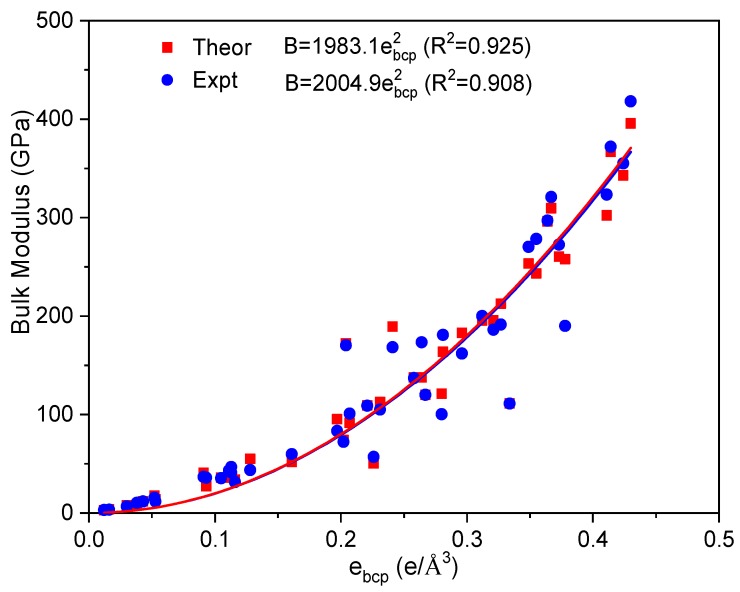
The relationship between *e_bcp_* and the bulk modulus of metals in ground state.

Figure 2 shows the relationship between *e_bcp_* and bulk modulus. The black and purple dots are the calculated and experimental values, respectively. By fitting bulk modulus against *e_bcp_* with a parabolic function, a relationship between them was obtained, as in Equation (2), with a coefficient, *k*_1_, around 2000 (GPa). The fitting curves overlap very well.

(2) $$B=k_{1}e_{bcp}^{2}$$

It is worth noting that the experimental bulk modulus is temperature dependent. Theoretically, one of methods to account the temperature effect to improve the calculation accuracy is to consider the zero-point energy and thermal phonon energy. Janthon et al. reported that the temperature effects on the lattice parameter, cohesive energy, and bulk modulus of transition metals are about 0.003−0.022 Å, 0.01−0.06 eV, and 1−17 GPa [37], respectively. We also estimate the difference between the calculated and experimental values of atomic volume and cohesive energy, as shown in Figure 1 and Figure 2. We used the experimental values of the equilibrium parameters in order to obtain more accurate correlations between *e_bcp_* and equilibrium properties. However, the resulted fitting parameters are close using calculated and experimental data, as shown in Figure 2. To explore the possible relationship between *e_bcp_* and equilibrium properties of metals, we have calculated the electronic parameters and equilibrium properties of 24 binary compounds as shown in Figure S1 and Table S1. There is linear relationship between their bulk modulus and *e_bcp_*.

**Figure 3:**
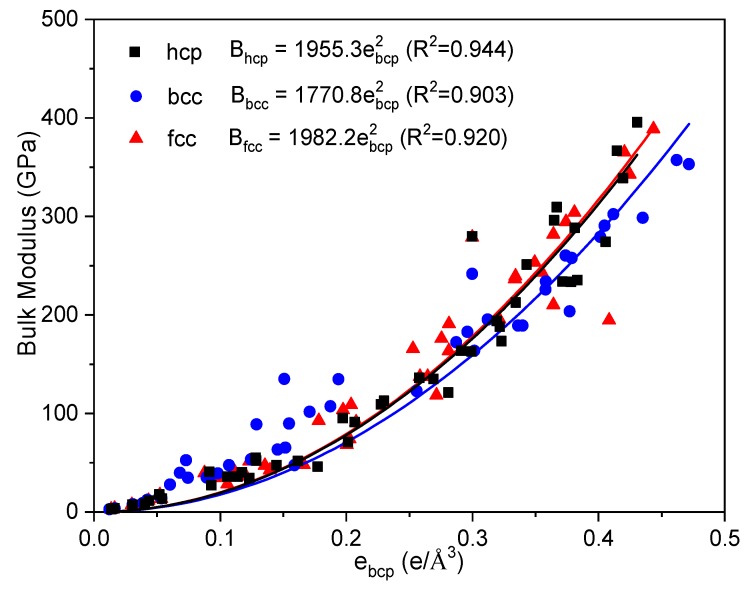
The relationship between the bulk modulus and *e_bcp_* of metals with hcp, bcc, and fcc structures.

To further clarify the influence of crystal structure, common crystal structures (hcp, bcc, and fcc,) are employed to check and extend the empirical relationship between *e_bcp_* and bulk modulus of metals and listed in Table 2. It should point out that, for specific metal, the crystal structure of its ground state is affirmatory; the other two structures are hypothetical at the same equilibrium conditions. As experimental bulk modulus is measured in the ground state of metals, here we used the theoretical values of bulk modulus from Ref. [5]. Figure 3 shows *e_bcp_* and bulk modulus of metals in hcp, bcc, and fcc structures. The black, blue, and red dots denote the bulk moduli for the hcp, bcc, and fcc structures, respectively. Generally, the parabolic relationships between *e_bcp_* and bulk modulus of metals present in Figure 3. Although the fitting for bcc structure (purple line) is somehow a bit separation than other two structures, the relationship between the numbers of electrons at the bcp and bulk modulus of metals approximately obey the expression of Equation (2) with a mean squared error around 0.92, as shown in Figure 3, building a connection between the macro bulk modulus and micro electron numbers. 

**Figure 4:**
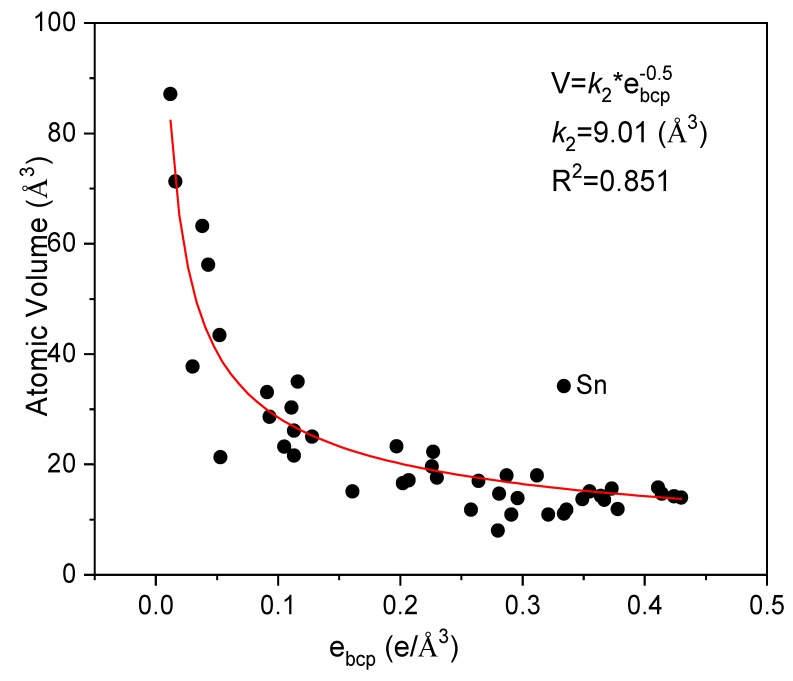
The relationship between atomic volume of metal and *e_bcp_*.

**Figure 5:**
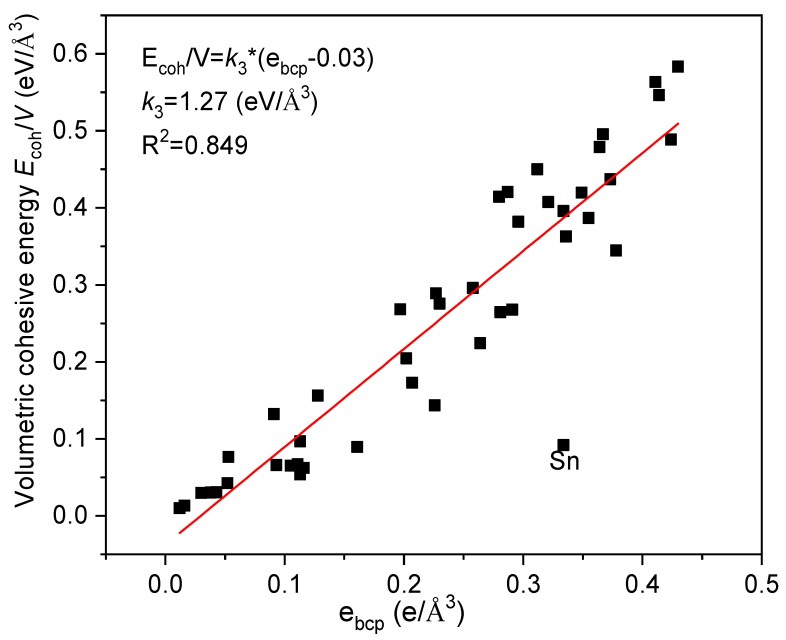
The relationship between the volumetric cohesive energy and *e_bcp_*.

**Figure 6:**
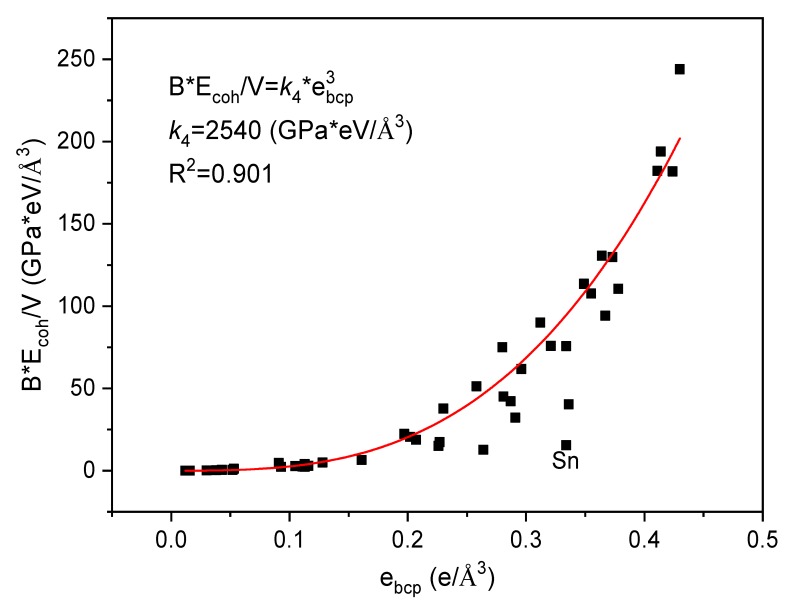
The relationship between the *B*·*E_coh_*/*V* and *e_bcp_*.

Atomic volume is a characteristic parameter of materials. It has been illustrated that there is a strong correlation between the bulk modulus and atomic volume of pure metals [5]. Here, we further explore the relationship between *e_bcp_* and atomic volume of metals. Figure 4 presents the relationship of the experimental values of atomic volume against *e_bcp_*. It could be fitted via an equation of

(3) $$V=k_{2}e_{bcp}^{-1/2}$$

where the coefficient *k*_2_ equals to 9.01 Å^3^. It is noted that point of metal Sn does not quite match Equation (3). It may be mainly because metal Sn has a diamond cubic crystal structure with relatively larger atomic volume. This phenomenon also appears in following volume related fittings (Figure 5 and Figure 6).

The bulk modulus is the second derivative of cohesive energy to volume. Therefore, many correlations exist among the cohesive energy, *E_coh_*, volume, *V*, and bulk modulus, *B*. However, the relationship between *E_coh_* and *B* of metals has not been studied directly. The volumetric cohesive energy *E_coh_*/*V* denotes the average cohesive energy of crystal, which has correlations with the bulk modulus and hardness [23]. It is interesting to explore the relationship between *E_coh_*/*V* and *e_bcp_* for metals due to the *e_bcp_* shows strong correlation with bulk modulus (Equation (2)). Figure 5 clearly shows a monotonic characteristic between *e_bcp_* and *E_coh_*/*V*, which delivers a linear relationship of

(4) $$E_{coh}/V=k_{3}(e_{bcp}-0.03)$$

where *k*_3_ = 1.27 eV/Å^3^.

As both *E_coh_*/*V* and *B* have correlated with *e_bcp_* (Figure 2 and Figure 5), the relationship between the *e_bcp_* and the products of *E_coh_*/*V* and *B* might be expected. Figure 6 shows the relationship between *B*·*E_coh_*/*V* and *e_bcp_*. The value of *B*·*E_coh_*/*V* fitted well with the value of *e_bcp_*, the relationship between them is very clear, which can be described by:

(5) $$\frac{BE_{coh}}{V}=k_{4}e_{bcp}^{3}$$

with *k*_4_ = 2540 (GPa*eV/Å^3^).

From above analysis, *e_bcp_* communicates the relationships of the equilibrium properties, such as bulk modulus (*B*), atomic volume (*V*), and *E*_coh_/*V*. Furthermore, if one of above parameters is known, others can be estimated through the bridge of *e_bcp_*, as shown in Table 3. The equations that are shown in Table 3 are derived from Equations (2) to (5). It is very convenient to estimate the equilibrium properties of simple metals. Due to the relationships between the volume and the structural parameters are definitely for the simple metals, the structural parameters can be derived from atomic volume. Furthermore, the atomic volume of metal strongly correlates with other parameters, such as *e_bcp_*, *E_coh_*/*V*, and *B*. Therefore, the structural parameters of simple metals correlate with the four equilibrium parameters *e_bcp_*, *E_coh_*, *V*, and *B*.

Some studies have built relationships between the electronic parameters and equilibrium parameters of metals. Shang et al. fitted a relationship between the bulk modulus and atomic volume *B* = 20,422*V*^−1.868^ [5]. Miedema et al. introduced an empirical relationship of *n_WS_* = 0.82 × (*B*/*V*)^1/2^ [16]. Cheng et al. reported a similar relationship [17]. Dolocan et al. derived a correlation of *B* = −Δ*E_coh_*/V, in which the Au, Cd, Ir, Pb, Pd, Pt, Re, Ru, and Zn have ∆ > 4 and the Ce, Cs, Gd, K, Li, Rb, and Zr have ∆ < 2 [38]. Tal proposed a direct relation between the charge density of a free atom, *ρ*, and the cohesive energy of the corresponding metal, *E_coh_* ~ *ρ*^5/3^, the bulk moduli of metals are also proportional to *ρ*^5/3^, however they do not work well for small values of cohesive energy and bulk modulus [39]. Our derived bulk modulus *B* is proportional to V^−4^, (*E_coh_*/*V*)^2^, and *e_bcp_*^2^. They are similar with reported relationships, except the larger weight of volume in our derived relationships.

## 4. Conclusions

First principles calculations have been taken to study the equilibrium properties of pure metals in this work. The equilibrium properties of metals, such as *V* and *E_coh_*, have been estimated based on first principles total energy calculation and an electronic structure parameter, the number of electrons at the bonding critical point, *e_bcp_*, was evaluated under AIM theory. In the present work, this parameter acts as the bridge communicating with the equilibrium properties of metals. The relationships between *e_bcp_* and equilibrium properties have been obtained through the fitting equilibrium properties against *e_bcp_*. Strong correlations have been achieved in these fittings. The bulk modulus obeys a parabolic relationship with *e_bcp_*, *E_coh_*/*V* linearly follows the variation of *e_bcp_*, and *V* varies via *e_bcp_* in the manner of $\propto e_{bcp}^{-0.5}$. This work builds a connection between the macro properties of metals and their micro bonding characteristics and provides a new way to estimate the bulk modulus (*B*) and volumetric cohesive energy (*E_coh_*/*V*) of pure metals.

## Figures and Tables

**Table 1:** Values of valence electrons *Z*, *E_coh_*, *V*, *e_bcp_*, and *B* of pure metals in ground state.

Metals	*Z*	*E_coh_* (eV/atom)		*V* (Å^3^/atom)			*e_bcp_* (e/Å^3^)	*B* (GPa)	
		Present	Exp **	Present	Shang *	Exp **	Present	Shang *	Exp **
Li^b^	3	1.59	1.63	20.4	20.3	21.3	0.053	13.9	11.6
Na^b^	9	1.05	1.113	36.9	37.1	37.7	0.030	7.9	6.8
K^b^	9	0.795	0.934	73.5	73.7	71.3	0.016	3.5	3.2
Rb^b^	9	0.682	0.852	91.2	90.8	87.1	0.012	2.7	3.1
Be^h^	2	3.69	3.32	7.9	7.9	8.01	0.280	121.1	100.3
Mg^h^	10	1.47	1.51	22.9	22.9	23.2	0.105	35.7	35.4
Ca^f^	10	1.86	1.84	42.6	41.8	43.4	0.052	17.4	15.2
Sr^f^	10	1.55	1.72	53.9	53.9	56.2	0.043	11.8	11.6
Ba^b^	10	1.79	1.90	62.3	62.5	63.2	0.038	9.0	10.3
Sc^h^	11	4.26	3.90	24.0	24.5	25.0	0.128	54.9	43.5
Ti^h^	12	5.56	4.85	17.1	17.3	17.6	0.230	112.8	105.1
V^b^	13	5.30	5.31	13.2	13.5	13.9	0.290	182.9	161.9
Cr^b^	14	4.03	4.10	11.4	11.6	11.9	0.378	257.7	190.1
Mn^c^	13	3.91	2.92	10.7	--	10.9	0.291	120.0	120.0
Fe^b^	14	4.49	4.28	10.5	11.4	11.8	0.336	189.3	168.3
Co^h^	15	4.92	4.39	10.3	10.9	11.1	0.334	212.5	191.4
Ni^f^	16	4.77	4.44	10.7	10.9	10.9	0.321	195.6	186.0
Cu^f^	17	3.47	3.49	12.0	12.0	11.8	0.258	137.5	137.0
Zn^h^	12	1.12	1.35	15.1	15.4	15.1	0.161	51.8	59.8
Y^h^	11	4.24	4.37	32.6	32.7	33.1	0.091	40.8	36.6
Zr^h^	12	6.39	6.25	23.3	23.4	23.3	0.197	95.3	83.3
Nb^b^	13	6.86	7.57	18.3	18.3	18.0	0.287	172.3	170.2
Mo^b^	14	6.33	6.82	15.7	16.0	15.6	0.373	260.4	272.5
Tc^h^	15	6.97	6.85	14.5	14.6	14.3	0.364	296.1	297.0
Ru^h^	16	7.06	6.74	13.9	13.9	13.6	0.367	309.4	320.8
Rh^f^	15	5.96	5.75	14.2	14.2	13.7	0.349	253.4	270.4
Pd^f^	16	3.72	3.89	15.4	15.5	14.7	0.281	163.7	180.8
Ag^f^	17	2.52	2.95	17.9	18.0	17.1	0.207	91.3	100.7
Cd^h^	18	0.764	1.16	22.4	23.0	21.6	0.113	35.8	46.7
Hf^h^	26	6.56	6.44	22.2	22.4	22.3	0.227	109.1	109.0
Ta^b^	27	8.28	8.10	18.1	18.3	18.0	0.312	195.3	200.0
W^b^	26	8.41	8.90	16.0	16.2	15.8	0.411	302.2	323.2
Re^h^	27	7.82	8.03	15.0	15.0	14.7	0.414	366.8	372.0
Os^h^	28	8.45	8.17	14.4	14.4	14.0	0.430	395.5	418.0
Ir^f^	29	7.55	6.94	14.6	14.6	14.2	0.424	342.8	355.0
Pt^f^	30	5.48	5.84	15.8	15.8	15.1	0.355	243.4	278.3
Au^f^	17	3.03	3.81	18.2	18.2	17.0	0.264	137.6	173.2
Al^f^	9	3.43	3.39	16.6	16.6	16.6	0.202	74.3	72.2
Ga^c^	13	2.68	2.81	20.4	--	19.6	0.226	50.3	56.9
In^c^	13	2.36	2.52	27.6	--	26.1	0.113	37.4	41.1
Tl^h^	13	2.08	1.88	30.9	31.3	28.6	0.093	27.2	35.9
Sn^c^	14	3.16	3.14	36.7	--	34.2	0.334	111.0	111.0
Pb^f^	14	2.93	2.03	31.7	31.9	30.3	0.111	40.6	43.0
Bi^c^	15	2.58	2.18	36.8	--	35.0	0.116	34.0	31.5

* Ref. [5]; ** Ref. [35]; The superscripts, ^b^, ^f^, ^h^, and ^c^ stand for the bcc, fcc, hcp, and cubic structures, respectively.

**Table 2:** Calculated *e_bcp_* and theoretical bulk modulus of pure metals with hcp, bcc, and fcc structures.

Metal.	*e_bcp_* (e/Å^3^)			*B* * (GPa)		
	hcp	bcc	fcc	hcp	bcc	fcc
Li	0.0539	0.0535	0.0534	13.5	13.9	13.5
Na	0.0304	0.0306	0.0301	7.6	7.9	7.5
K	0.0163	0.0165	0.0163	3.5	3.5	3.5
Rb	0.0138	0.0122	0.0136	2.7	2.7	2.7
Be	0.2809	0.2558	0.2714	121.1	122.9	118.5
Mg	0.1059	0.0892	0.0979	35.7	34.9	34.7
Ca	0.0518	0.0520	0.0523	17.7	16.0	17.4
Sr	0.0435	0.0424	0.0431	11.4	12.2	11.8
Ba	0.0401	0.0388	0.0390	8.4	9.0	8.3
Sc	0.1282	0.1244	0.1232	54.9	53.2	51.8
Ti	0.2300	0.1874	0.2037	112.8	107.3	109.0
V	0.3232	0.2900	0.2755	173.2	182.9	176.0
Cr	0.3782	0.3788	0.3337	233.5	257.7	236.7
Mn	0.2998	0.2998	0.2998	279.7	241.8	278.9
Fe	0.3810	0.3362	0.4084	288.3	189.3	194.6
Co	0.3343	0.3770	0.3641	212.5	203.6	210.2
Ni	0.3194	0.3399	0.3214	193.8	189.1	195.6
Cu	0.2580	0.1939	0.2584	136.1	134.6	137.5
Zn	0.1616	0.1456	0.2004	51.8	63.3	68.4
Y	0.0915	0.0981	0.0876	40.8	39.3	39.7
Zr	0.1972	0.1546	0.1781	95.3	89.7	92.9
Nb	0.2992	0.2871	0.2529	162.8	172.3	165.7
Mo	0.3713	0.3738	0.3342	233.8	260.4	239.4
Tc	0.3648	0.4048	0.3740	296.1	290.6	294.7
Ru	0.3670	0.4015	0.3809	309.4	279.1	304.2
Rh	0.3431	0.3581	0.3493	251.1	225.9	253.4
Pd	0.2912	0.3016	0.2813	163.6	163.5	163.7
Ag	0.2067	0.1289	0.2078	91.1	88.9	91.3
Cd	0.1139	0.0920	0.1395	35.8	35.8	42.6
Hf	0.2274	0.1709	0.1974	109.1	101.8	103.6
Ta	0.3216	0.3122	0.2815	188.0	195.3	191.2
W	0.4057	0.4118	0.3641	274.3	302.2	281.9
Re	0.4146	0.4619	0.4205	366.8	357.2	365.2
Os	0.4305	0.4717	0.4435	395.5	353.0	388.8
Ir	0.4193	0.4351	0.4247	339.0	298.6	342.8
Pt	0.3832	0.3583	0.3550	235.2	233.8	243.4
Au	0.2691	0.1508	0.2646	135.0	134.9	137.6
Al	0.2014	0.1518	0.2024	70.8	65.2	74.3
Ga	0.1773	0.1070	0.1664	45.9	47.4	48.0
In	0.1231	0.0745	0.1206	34.4	34.7	36.2
Tl	0.0931	0.0603	0.1057	27.2	27.9	28.4
Sn	0.1448	0.1590	0.1355	47.6	47.5	47.2
Pb	0.1173	0.0682	0.1118	40.2	39.8	40.6
Bi	0.1286	0.0730	0.1269	52.0	52.6	51.9

* Ref. [5].

**Table 3:** Empirical relationships between *e_bcp_* (e/Å^3^), atomic volume of metal, *V* (Å^3^), bulk modulus, *B* (GPa), and volumetric cohesive energy, *E_coh_*/*V* (eV/Å^3^).

	*e_bcp_*	*V*	*B*	*E_coh_*/*V*
*e_bcp_*	--	(*k*_2_/*V*)^2^	*B*^0.5^/*k*_1_	*E_coh_*/(*Vk*_3_) + 0.03
*V*	$k_{2}e_{bcp}^{-0.5}$	--	$k_{2}k_{1}^{0.25}B^{-0.25}$	$k_{2}{\left(\frac{E_{coh}}{k_{3}V}+0.03\right)}^{-0.5}$
*B*	$k_{1}e_{bcp}^{2}$	$k_{1}k_{2}^{4}V^{-4}$	--	$k_{1}{\left(\frac{E_{coh}}{k_{3}V}+0.03\right)}^{2}$
*E_coh_*/*V*	*k*_3_(*e_bcp_* − 0.03)	$k_{3}(k_{2}^{2}V^{-2}-0.03)$	$k_{3}(k_{1}^{-0.5}B^{0.5}-0.03)$	--

*k*_1_ = 2005 GPa obtained by fitting experimental bulk modulus; *k*_2_ = 9.01 Å^3^; *k*_3_ = 1.27 eV/Å^3^.

## Supplementary Materials

The following are available online at https://www.mdpi.com/1996-1944/12/18/2932/s1, Figure S1: The relationship between bulk modulus and *e_bcp_* of binary compounds. Table S1: Values of *E_coh_*, *V*, *e_bcp_*, and *B* of alloys in ground state.
