# Supplementary material for: Correlations of Equilibrium Properties and Electronic Structure of Pure Metals
Source: Materials (Basel). 2019 Sep 11;12(18):2932. doi: 10.3390/ma12182932 (PMC6766288; doi:10.3390/ma12182932)
Supplement: Supplementary file 1 [file materials-12-02932-s001.pdf]

materials

MDPI

Article

Correlations of equilibrium properties and electronic structure of pure metals

J. H. Dai [1], D. Y. He [2,*] and Y. Song [1,*]

[1] School of Materials Science and Engineering, Harbin Institute of Technology at Weihai, 2 West Wenhua Road, Weihai 264209, China
[2] School of Materials Science and Engineering, Jilin University, 5988 Renmin Street, Changchun 130025, China
* Corresponding author: hedy@jlu.edu.cn (D.Y.H.), sy@hitwh.edu.cn (Y.S.); Tel.: xxxx

Received: 09 August 2019; Accepted: 09 September 2019; Published: date

To explore the possible relationship between e_{bcp} and equilibrium properties of metals, we further calculated the electronic parameters and equilibrium properties of 24 binary alloys including AB and A_3B (or AB_3) type alloys (Table S1). A parabolic relationship between bulk modulus and e_{bcp} for A_3B (or AB_3) type alloys was obtained as illustrated in Figure S1(a), but not for the AB type alloys, which shows a roughly linear relationship between bulk modulus and e_{bcp} with a mean squared error of 0.818 (Figure S1(b)). However, no strict correlation between e_{bcp} and cohesive energy or atomic volume was found.

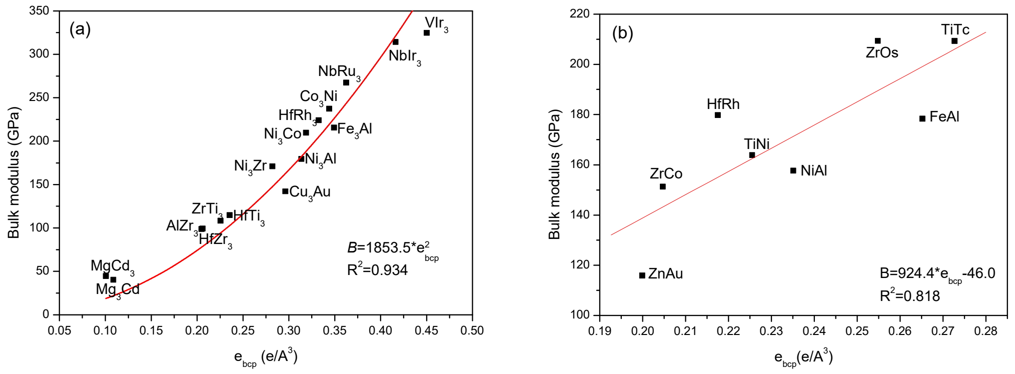

Figure S1. The relationship between bulk modulus and e_{bcp} of (**a**) A_3B (or AB_3) type and (**b**) AB type binary alloys.

Table S1. Values of E_{coh}, V, e_{bcp}, and B of alloys in ground state, and their crystal structures.

Alloys	Structure	e_{bcp} (e/Å³)	B (GPa)	V (Å³)	E_{coh} (eV)
AlFe	B2	0.2652	178.36	23.61	5.67
AlNi	B2	0.2351	157.66	24.21	4.98
HfRh	B2	0.2175	179.76	34.63	7.40
TiNi	B2	0.2255	163.83	27.10	6.04
TiTc	B2	0.2727	209.33	29.61	8.46
ZnAu	B2	0.1999	115.87	32.40	2.31
ZrCo	B2	0.2047	151.31	32.04	6.98
ZrOs	B2	0.2548	209.39	35.37	9.17
$AlFe_3$	$L1_2$	0.3493	215.49	44.72	6.17
$AlNi_3$	$L1_2$	0.3136	179.53	45.32	5.14

Cu_3Au	$L1_2$	0.2961	141.99	54.17	3.52
$HfRh_3$	$L1_2$	0.3325	223.97	60.79	7.08
$NbIr_3$	$L1_2$	0.4163	314.17	60.32	9.13
$NbRu_3$	$L1_2$	0.3626	267.28	58.83	8.70
VIr_3	$L1_2$	0.4503	324.77	56.23	8.65
Zr_3Al	$L1_2$	0.2060	99.22	84.09	6.34
Co_3Ni	$D0_{19}$	0.3440	237.25	83.64	5.92
$CoNi_3$	$D0_{19}$	0.3187	209.64	85.85	5.30
$HfTi_3$	$D0_{19}$	0.2355	114.68	147.50	6.45
$HfZr_3$	$D0_{19}$	0.2047	98.53	184.87	6.90
Mg_3Cd	$D0_{19}$	0.1087	40.31	175.26	1.39
$MgCd_3$	$D0_{19}$	0.1006	44.41	175.88	0.99
$ZrNi_3$	$D0_{19}$	0.2821	170.85	105.52	6.02
$ZrTi_3$	$D0_{19}$	0.2255	108.23	149.61	6.42
